# Vaccination in Relation to Blindness

**Published:** 1856-10

**Authors:** 


*Vaccination in relation to Blindness.*

Statistical researches show us that, prior to Jenner's discovery, of 100 cases of blindness, 55 were due to small-pox; and

Dr. Dumont, physician to the Hospice for the Blind, has recently supplied an interesting account of the progressive decrease of that proportion. Among the blind of 60 years of age, he finds this variety of cause in 12 per cent; in adults, it only exists as 8 per cent.; and, in children, only as 5 per cent. We may take as a mean, counting all ages, about 7 per cent., which, as at the commencement of the present century, the proportion was 35 per cent., exhibits a diminution of 28 per cent.—*New York Journal of Medicine.*

